# Overcoming the Low Oral Bioavailability of Deuterated Pyrazoloquinolinone Ligand DK-I-60-3 by Nanonization: A Knowledge-Based Approach

**DOI:** 10.3390/pharmaceutics13081188

**Published:** 2021-07-31

**Authors:** Jelena R. Mitrović, Branka Divović-Matović, Daniel E. Knutson, Jelena B. Đoković, Aleksandar Kremenović, Vladimir D. Dobričić, Danijela V. Randjelović, Ivana Pantelić, James M. Cook, Miroslav M. Savić, Snežana D. Savić

**Affiliations:** 1 Department of Pharmaceutical Technology and Cosmetology, Faculty of Pharmacy, University of Belgrade, Vojvode Stepe 450, 11221 Belgrade, Serbia; jelena.mitrovic@pharmacy.bg.ac.rs (J.R.M.); jelena.djokovic@pharmacy.bg.ac.rs (J.B.Đ.); ivana.pantelic@pharmacy.bg.ac.rs (I.P.); 2 Department of Pharmacology, Faculty of Pharmacy, University of Belgrade, Vojvode Stepe 450, 11221 Belgrade, Serbia; branka.divovic@pharmacy.bg.ac.rs (B.D.-M.); miroslav.savic@pharmacy.bg.ac.rs (M.M.S.); 3 Department of Chemistry and Biochemistry, Milwaukee Institute for Drug Discovery, University of Wisconsin-Milwaukee, 3210N. Cramer St., Milwaukee, WI 53211, USA; knutsond@uwm.edu (D.E.K.); capncook@uwm.edu (J.M.C.); 4 Laboratory of Crystallography, Faculty of Mining and Geology, University of Belgrade, Đušina 7, 11000 Belgrade, Serbia; aleksandar.kremenovic@rgf.bg.ac.rs; 5 Department of Pharmaceutical Chemistry, Faculty of Pharmacy, University of Belgrade, Vojvode Stepe 450, 11221 Belgrade, Serbia; vladimir.dobricic@pharmacy.bg.ac.rs; 6 Department of Microelectronic Technologies, Institute of Chemistry, Technology and Metallurgy, University of Belgrade, Njegoševa 12, 11000 Belgrade, Serbia; danijela@nanosys.ihtm.bg.ac.rs

**Keywords:** pyrazoloquinolinones, nanocrystals, wet media milling, fasted/fed bioavailability

## Abstract

Poor water solubility of new chemical entities is considered as one of the main obstacles in drug development, as it usually leads to low bioavailability after administration. To overcome these problems, the selection of the appropriate formulation technology needs to be based on the physicochemical properties of the drug and introduced in the early stages of drug research. One example of the new potential drug substance with poor solubility is DK-I-60-3, deuterated pyrazoloquinolinone, designed for the treatment of various neuropsychiatric disorders. In this research, based on preformulation studies, nanocrystal technology was chosen to improve the oral bioavailability of DK-I-60-3. Nanocrystal dispersions stabilized by sodium lauryl sulfate and polyvinylpyrrolidone were prepared by modified wet media milling technique, with the selection of appropriate process and formulation parameters. The nanoparticles characterization included particle size and zeta potential measurements, differential scanning calorimetry, X-ray powder diffraction, dissolution and solubility study, and in vivo pharmacokinetic experiments. Developed formulations had small uniform particle sizes and were stable for three months. Nanonization caused decreased crystallite size and induced crystal defects formation, as well as a DK-I-60-3 solubility increase. Furthermore, after oral administration of the developed formulations in rats, two to three-fold bioavailability enhancement was observed in plasma and investigated organs, including the brain.

## 1. Introduction

The emerging trends in the combinatorial chemistry and design of psychopharmacological drugs led to the development of drug candidates with increased lipophilicity and high molecular weight. It is estimated that 90% of new chemical entities are poorly soluble, which is recognized as a major obstacle in drug development [1]. Because conventional formulations fail to meet requirements for successful pharmacology studies in the preclinical investigation, the role of the formulation scientist becomes crucial to implement advanced formulation strategies early in development processes [2]. However, many attempts underachieve to overcome poor solubility due to the selection of formulation strategy driven by researchers’ experience rather than physicochemical properties of the drug substance [3]. Understanding the relation between features of the active ingredient, as well as special aspects of the formulations and the required in vivo performance, is necessary to select the optimal formulation approach and minimize the risk of failures in clinical trials due to insufficient or highly variable drug exposures [4].

By evaluating basic information on molecular structure, molecular weight, as well as results from preformulation studies such as solubility in water and oils, melting temperature, crystallinity, and amorphization ability, formulation steps can be determined using a knowledge-based approach. Generally, poorly soluble substances can be characterized as brick dust or grease balls depending on the limiting factor for the solubility: solid-state or solvation process [3,5]. Brick dust substances are poorly soluble in an aqueous environment as well as in lipids and organic solvents, and therefore represent ideal candidates for nanosizing, especially if amorphization is not possible [1]. Nanosizing or nanonization implies particle size reduction to the sub-micron range, thus obtaining nanocrystal dispersions [6].

High drug loading, relatively simple composition, and easy production make nanocrystal dispersions an interesting option for many challenging drug substances [7], especially the ones with solubility in water less than 200 µg/mL, high melting point, and high molecular weight [8]. Although suitable for almost all administration routes, the majority are investigated for oral administration. Many drugs containing nanocrystals intended for oral administration reached the market to this date, with an increasing number in preclinical and clinical trials [6,9]. Because of small particle size, solubility, dissolution, and adhesiveness to membranes are improved. Owing to these distinctive features, higher drug concentrations and bioavailability after oral administration are expected, with reduced food effects [7,10]. Because absorption of poorly soluble drugs can be altered in different prandial states, designing a formulation resistant to food effects early in drug development is beneficial. Additionally, it can provide a commercial advantage and prevent costly reformulation later in the product lifecycle [11].

Deuterated pyrazoloquinolinones are investigated as non-benzodiazepine ligands to interact with α6β2/3γ2 subunit of GABAA receptors with improved metabolic stability to retard O-demethylation. Deuteration of both C4′ and C7′ methoxy groups in the parent molecule resulted in the synthesis of the ligand DK-I-60-3 (7-Methoxy-d3-2-(4-methoxy-d3-phenyl)-2,5-dihydro-3Hpyrazolo[4,3-c]quinolin-3-one). Extensive research published in Knutson et al. [12] proved its efficacy and selectivity, as well as metabolic stability, and made it one of the candidates for the treatment of various neuropsychiatric disorders. However, very low solubility could jeopardize its further investigation in in vivo preclinical studies. So far, the examination of DK-I-60-3 was focused on toxicology and pharmacology studies, with no data on its physicochemical properties.

Therefore, we aimed through this research to select a formulation strategy based on physicochemical properties of DK-I-60-3 in order to enhance bioavailability after oral administration. As a first step, preformulation studies were designed to obtain insight into the key properties of DK-I-60-3, which would guide further stages of formulation development. Afterward, during nanocrystal preparation, an investigation of formulation and process parameter effects on particle size was conducted to obtain dispersions with small and uniform particle size and satisfactory short-term stability. Detailed physicochemical characterization was performed in order to obtain a closer insight into the morphology, physical state, solubility, and dissolution of developed formulations. Finally, in vivo pharmacokinetic study in rats tested nanocrystals’ ability to improve the bioavailability after oral administration taking into account different prandial states.

## 2. Materials and Methods

### 2.1. Materials

DK-I-60-3 was synthesized at the Department of Chemistry and Biochemistry, University of Wisconsin—Milwaukee, WI, USA, according to the procedure explained by Knutson et al. [12]. Other materials used in this research are listed: medium-chain triglycerides, propylene glycol (Fagron GmbH & KG, Barsbüttel, Germany), soybean oil (Lipoid Purified Soybean Oil 700, Lipoid GmbH, Ludwigshafen, Germany), castor oil, isopropanol, benzyl alcohol, polyethylene glycol 400, sodium lauryl sulfate (Sigma-Aldrich Laborchemikalien GmbH, Seelze, Germany), polyvinylpyrrolidone K25 (Carl Roth GmbH + Co. KG, Karlsruhe, Germany) methanol, ethanol, and dimethyl sulfoxide (Fisher Scientific, Loughborough, UK). Fresh ultrapure water was supplied from a TKA GenPure system (TKA Wasseranfbereitungssysteme GmbH, Neiderelbert, Germany).

### 2.2. Methods

#### 2.2.1. Preformulation Studies

##### Solubility of DK-I-60-3

Solubility of DK-I-60-3 in distilled water, 0.1 M hydrochloride acid, phosphate buffer (pH 6.8), benzyl alcohol, polyethylene glycol 400, commonly used oils (medium-chain triglycerides, soybean oil, and castor oil), and organic solvents (isopropanol, methanol, ethanol, and dimethyl sulfoxide) at 25 °C was investigated by the shake flask method. An excess amount of DK-I-60-3 was added to investigated solvents and vortexed for 24 h. Samples were then centrifuged, supernatant diluted by isopropanol, and DK-I-60-3 concentration determined by LC-MS/MS.

##### Melt-Quenching

For this analysis, around 2 mg of the unprocessed substance was heated to 330 °C, with a heating rate of 20 °C/min, then cooled to 25 °C with a cooling rate of 40 °C/min and finally heated again to 330 °C with a heating rate of 10 °C/min. The measurements were conducted under nitrogen flow (50 mL/min) by DSC 1 (Mettler-Toledo AG, Analytical, Greifensee, Switzerland). The onset and peak temperature, and enthalpy were evaluated by STAR^®^ SW 12.10 software.

#### 2.2.2. Nanocrystal Dispersions Preparation

For nanocrystal dispersions preparation, a modified wet ball milling technique was applied, similarly to the procedure explained earlier [13]. DK-I-60-3 (0.2% *w/w*) was homogenized in aqueous stabilizer solution for 5 min at 10,000 rpm on a rotor stator homogenizer IKA Ultra-Turrax^®^ T25 digital (IKA^®^-Werke GmbH & Company KG, Staufen, Germany). Eight formulations with different sodium lauryl sulfate (SLS) and polyvinylpyrrolidone (PVP) concentrations were prepared. The concentration of SLS was 0.02% (F1–F4), or 0.05% (F5–F8), while the SLS:PVP ratio was varied: 1:10, 1:4, 1:2 or 1:1 (Table 1). Obtained dispersion was then transferred to 2 mL tubes containing 20%, 40%, or 60% *v*/*v* 0.1–0.2 mm yttrium stabilized zirconium beads (SiLibeads^®^ Typ ZY-S, Sigmund Lindner GmbH, Warmensteinach, Germany) and shaken for 0.5 h or 1 h using a beads-milling cell disruptor equipment (Disruptor Genie, Scientific Industries, Bohemia, NY, USA). After the milling, nanocrystal dispersion was separated from beads and stored in glass bottles for two months. The concentration of DK-I-60-3 in dispersions was determined by LC-MS/MS after diluting 10 µL of the dispersion in 10 mL of isopropanol.

#### 2.2.3. Physicochemical Characterization

For the characterization, as reference formulations, coarse suspension (containing DK-I-60-3 in water in the same concentration as in nanocrystal dispersions) and corresponding suspensions S5, S6, and S8 (with the same qualitative and quantitative composition as F5, F6, and F8, respectively) were prepared by mixing the components on the magnetic stirrer (RH basic 2 IKAMAGR Magnetic Stirrer; IKAR-Werke GmbH & Company KG).

##### Particle Size Measurements

The particle size and particle size distribution expressed as the mean hydrodynamic diameter (z-ave) the polydispersity index (PDI) were determined by dynamic light scattering (DLS) technique on the Zetasizer Nano ZS90 (Malvern Instruments Ltd., Worcestershire, U.K.). Before measurements, nanocrystal samples were diluted in ultra-purified water (1:100, *v*/*v*).

##### Zeta Potential Measurements

The zeta potential (ZP) of obtained nanocrystal dispersions was determined by Zetasizer Nano ZS90 (Malvern Instruments Ltd., Worcestershire, U.K.) after dilution in ultrapure water (conductivity 50 μS/cm) 1:100 (*v*/*v*).

##### Atomic Force Microscopy (AFM)

Morphology and size of the nanocrystals in developed dispersions were analyzed using an NTEGRA Prima Atomic Force Microscope (NT-MDT, Moscow, Russia). The sample preparation included dilution of the dispersion F5, F6, or F8 in ultra-purified water (1:100 *v*/*v*), after which the 10 µL of the sample was placed on the circular mica substrate (Highest Grade V1 AFM Mica Discs, Ted Pella Inc., Redding, CA, USA) and dried in a vacuum dryer (30 min, 25 °C). The measurements were conducted using intermittent-contact AFM mode using NT-MDT NSGO1 silicon cantilevers (N-type, Antimony doped, Au reflective coating), with a nominal force constant of 5.1 N/m. The cantilever driving frequency was around 150 kHz during the measurements. For the analysis of the taken topography and “error signal” AFM images, the software Image Analysis 2.2.0 (NT-MDT) was used.

##### Differential Scanning Calorimetry (DSC)

Around 2 mg of the dried samples of nanocrystals or physical mixtures were heated to 330 °C in aluminum pans with a heating rate of 10 °C/min using DSC 1 (Mettler-Toledo AG, Analytical, Greifensee, Switzerland). The nitrogen flow during the measurements was set to 50 mL/min.

##### X-ray Powder Diffraction Analysis (XRPD)

Nanocrystal dispersions with different particle sizes, corresponding suspension, and unprocessed DK-I-60-3 were investigated by X-ray powder diffraction (XRPD). Dispersions were centrifuged, and the analysis was performed on dried sediment. The measurements were performed on a Rigaku Smartlab X-ray Diffractometer (Tokyo, Japan) in θ-θ geometry in parafocusing Bragg–Brentano geometry. The used detector was D/teX Ultra 250 strip detector in 1D standard mode with CuKα1,2 radiation source (U = 40 kV and I = 30 mA). The 2θ range in which the results were collected was from 3° to 40°, with the step of 0.01° (data collection speed was 3 °/min). To minimize the low background, a single crystal silicon sample holder was used. The XRPD data were treated by the PDXL2 integrated X-ray powder diffraction software (v. 2.8.30; Rigaku Corporation, Tokyo, Japan).

##### Dissolution Study

For the dissolution study, the direct dialysis bag method was used. Dialysis bag (cellulose membrane MW cut-off 12000, Sigma-Aldrich, St. Louis, MO, USA) was filled with 2 mL of nanocrystal dispersion (F5, F6 or F8), corresponding suspension (S5, S6, or S8), or coarse DK-I-60-3 suspension in water and immersed in 200 mL of dissolution medium (water:isopropanol 9:1, *v*/*v*). The test was performed at 37 °C. At predetermined time points (5 min, 20 min, 1 h, 2 h, 4 h, and 8 h), 1 mL of medium with dissolved particles was withdrawn and replaced with 1 mL of fresh medium. The experiments were performed in triplicate, and the concentration of DK-I-60-3 in samples was analyzed by the LC-MS/MS method.

##### Solubility of Nanocrystals

Solubility of nanocrystals in formulations F5, F6, and F8, corresponding suspensions (S5, S6, and S8) was determined at 37 °C. A total of 300 µL of the sample was added to 1.5 mL ultra-purified water, 0.1 M hydrochloride acid, or phosphate buffer (pH 6.8) and shook at 250 rpm for 24 h. Samples were centrifuged (14,000× *g*, MiniSpin^®^ plus centrifuge, Eppendorf, Hamburg, Germany), supernatants filtered through 0.22 µm syringe filters and diluted in isopropanol. The concentration of DK-I-60-3 was determined by the LC-MS/MS method.

#### 2.2.4. In Vivo Pharmacokinetic and Biodistribution Studies

Pharmacokinetic studies were conducted after oral administration of nanocrystal formulation F5 and corresponding coarse suspension with the same qualitative and quantitative content (S5) in fasted and fed state. In fasted group, food intake was restricted 10 h before and 4 h after formulation administration, while water was provided ad libitum. Male Sprague-Dawley rats weighing 150–200 g were housed six animals per cage. In the animal room, the light/dark period was set on 12 h cycles (light on at 6:00 a.m) with the illumination 120 lx. The temperature was set at 22 ± 1 °C, with the relative humidity 40%–70%. The research was conducted in accordance with the National Institutes of Health Animal Care and Use Committee guidelines. The study was approved by the Ethics Committee on Animal Experimentation of the University of Belgrade—Faculty of Pharmacy, Serbia and Ministry of agriculture, forestry and water management—Veterinary Directorate (323-07-13805/2020-05 from 31.12.2020)

Experimental animals were divided into four groups, each group containing 18 animals (three animals per time point): group I received F5 in fasted conditions, group II received F5 in fed conditions, group III received S5 in fasted conditions, and group IV received S5 in fed conditions. The test formulations were administered orally via a gastric probe in a volume of 5 mL/kg (the administered dose was 10 mg/kg). At predetermined time points (15 min, 1 h, 4 h, 8 h, 16 h, and 36 h) after administration, animals were anesthetized by ketamine hydrochloride (90 mg/kg, 10% Ketamidor, Richter Pharma AG, Wels, Austria). The samples of blood were collected via cardiac puncture in heparinized syringes and centrifuged for 10 min at 1000× *g* (MiniSpin^®^ plus centrifuge, Eppendorf, Hamburg, Germany) for plasma separation. Parts of the liver, kidney, and brain were taken as well, weighted, and homogenized in 1 mL of methanol by ultrasonic probe. Homogenized samples were then centrifuged for 20 min at 3400× *g*, and supernatants were separated. The DK-I-60-3 was extracted from plasma and supernatants by solid-phase extraction using Oasis HLB cartridges (Waters Corporation, Milford, MA, USA). The cartridges were preconditioned by methanol and water and then loaded with samples and internal standard solution, while the endogenous impurities were removed by washing the cartridges with water and methanol. The elution was carried out by 1 mL of methanol for 1 min. The concentration of DK-I-60-3 in the eluates was determined by LC-MS/MS method. From the concentration data, the pharmacokinetic parameters were calculated by “PK Functions for Microsoft Excel” (https://www.pharmpk.com/soft.html, accessed on 1 June 2021).

#### 2.2.5. Analytical Method

For the determination of DK-I-60-3 in all experiments, the liquid chromatography-tandem mass spectrometry (LC-MS/MS) method was used on the UHPLC chromatograph ACELLA (Thermo Fisher Scientific Inc., Madison, WI, USA), coupled to a triple quadrupole mass spectrometer TSQ Quantum Access MAX (Thermo Fisher Scientific Inc., Madison, WI, USA) with a heated electrospray ionization (HESI) interface. The samples were injected in a volume of 10 µL. The separation was performed on the XTerra MS C18 column (150 × 2.1 mm, 3.5 µm particle size). The mobile phase containing acetonitrile and 0.1% formic acid (50:50, *v*/*v*) was eluated at the flow rate of 0.3 mL/min. The column temperature was set to 35 °C. Detection and quantification of DK-I-60-3 and internal standard were performed in positive HESI mode (*m*/*z* = 328.10–282.05 and *m*/*z* = 349.00–303.85, respectively).

#### 2.2.6. Statistical Analysis

All measurements were performed in triplicate, and results were expressed as mean value ± SD, except in in vivo study where SEM was used instead of SD. After checking the normality of distribution, the statistical analysis involved Student’s *t*-test for two sets of results or ANOVA with Turkey HSD as a post hoc test for three results groups. Statistical analysis was performed using IBM SPSS Statistics software (v. 25). *p* < 0.05 was considered as statistically significant.

## 3. Results and Discussion

### 3.1. Preformulation Studies

**Figure 1:**
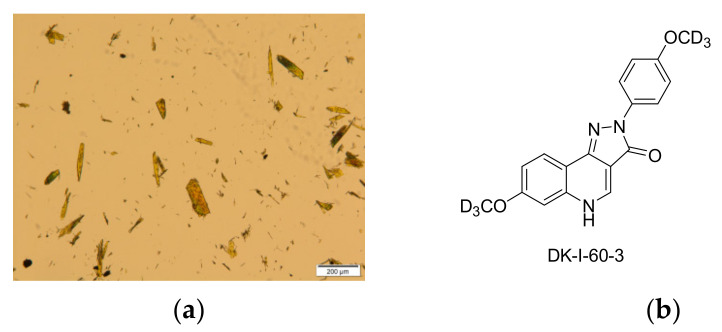
(**a**) Micrograph of coarse DK-I-60-3 powder; (**b**) chemical structure of DK-I-60-3.

Substance DK-I-60-3 appeared as a yellow crystalline powder, with broad particle size distribution from 2.15 to 191.16 µm (Figure 1a). The solubility of DK-I-60-3 was comparable to the solubility of monodeuterated analog DK-I-56-1 (Table 2) [13]. As expected, because of the absence of ionizable functional groups (Figure 1b), the solubility did not change under different pH conditions. Furthermore, solubility in oils was low, excluding the lipid formulations to incorporate DK-I-60-3. Results revealed that the solubility in organic solvents was low as well, around 1 mg/mL, except in dimethyl sulfoxide. Moreover, it was slightly soluble in polyethylene glycol 400, which is often used as a co-solvent in preclinical investigation, with or without surfactants. Being poorly soluble not only in water but also in lipid and organic solvents, DK-I-60-3 could be considered as a brick dust molecule. For substances with these characteristics, amorphization or nanocrystal technology would be the formulation strategy of choice to overcome their low solubility [1,3].

**Figure 2:**
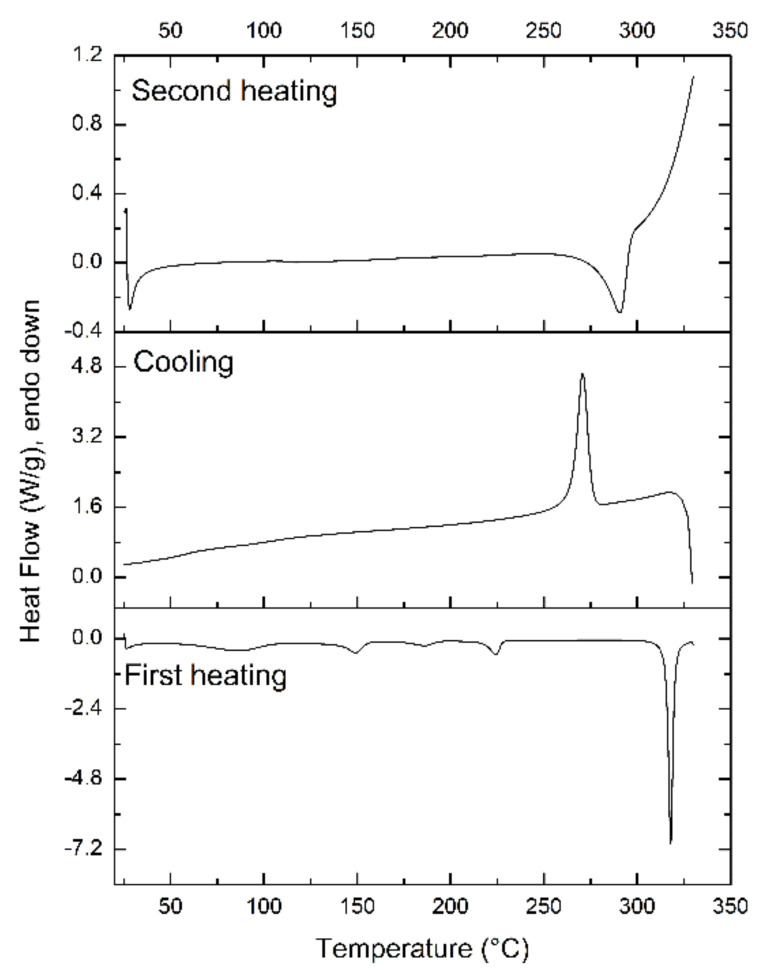
Thermograms showing the crystallization tendency of DK-I-60-3 after melt cooling.

However, not all substances are suitable candidates for amorphization. Based on the ability to vitrify upon cooling from the melted state or to form a glass, materials can be characterized as glass-formers or non-glass-formers. According to the results of prediction of glass-forming ability for more than 100 pharmaceutical ingredients published by Alhalaweh et al. [5], it could be speculated that DK-I-60-3 would not be stable in the amorphous form. Aromatic rings, as rigid structures, typically contribute to the denser packing of molecules in the crystal lattice and therefore increased crystallization tendency. To test this theory, the ability of the substance to become amorphous when cooled after melting was investigated by quenching the melt of DK-I-60-3 (Figure 2).

In the first heating cycle, DK-I-60-3 was completely melted (melting enthalpy 74.62 J/g). During the succeeding rapid cooling process, crystallization occurred shortly after the cooling started, which was indicated by an exothermic peak at 271 °C. In the second heating phase, a relatively broad endothermic peak at 290 °C (melting enthalpy 42.79 J/g) was observed and was attributed to the melting of the crystalized drug form. These results are typical for class I molecules with high crystallization tendency or non-glass-formers [14]. The crystallinity index calculated from the melting enthalpies of melting peaks during first and second heating was 57.34%. Lower crystallinity could be attributed to a partially disordered crystal lattice, also indicated by the peak broadening (difference between onset and peak temperature was 2.12 and 15.24 °C in first and second heating, respectively).

### 3.2. Nanocrystal Dispersions Preparation and Physicochemical Characterization

#### 3.2.1. Process Parameters

The results from preformulation studies clearly suggested that DK-I-60-3 was a suitable candidate for nanocrystal dispersions. It is well known that there are two main ways of producing nanocrystals: top-down and bottom-up. However, because of poor solubility in organic solvents, the bottom-up method was excluded in this case [15]. Among many top-down techniques, wet media milling is considered optimal early in pharmaceutical development. One of the most important process parameters that affect the kinetics of particle size decrease is milling duration [16]. According to particle size measurements right after preparation, milling time had a significant influence on z-ave and PDI in all formulations (*p* < 0.05, ANOVA) (Table 3 and Table S1). After its prolonging, the particle size decreased to 50 nm or more, regardless of milling media volume. PDI decreased after one hour of milling as well, suggesting that more uniform dispersion was obtained, although, in some formulations, the change was not pronounced. As the time increases, the chance for collision between milling media and DK-I-60-3 particles increases as well, so the breakage of the substance particles is more likely to occur. Furthermore, more time is available for the interaction between stabilizers and the drug substance. However, many papers suggest that after a certain time, the particle size achieves a constant level, and continuing the milling does not further decrease the particle size, probably because of the increased likelihood of interparticle collisions [8].

As the second process parameter in this research, the effects of zirconium beads amount (60%, 40%, and 20% of tube volume) on particle size and size distribution were investigated. The observed dependence was not straightforward. Considering the technological process, inside the tubes, three types of interaction are present: bead-DK-I-60-3, bead-bead, and DK-I-60-3-DK-I-60-3. For efficient nanonization, the collision between drug particles and grinding media must be dominant, which would be enabled by the addition of the proper amount of milling beads [8]. In fact, milling media volume had a significant impact on particle size in all formulations (*p* < 0.001, ANOVA), and the size differences were proportional to the beads volume differences, i.e., the effect was more pronounced in case of change from 20% to 40%. It can be noticed that after one hour of milling, with double beads volume increase (from 20% to 40%), particle size decreased for 30–60 nm, and by further volume increase to 60%, the size was reduced for an additional 20 nm in most formulations (Table 3).

Two-way ANOVA results showed that interaction between milling time and beads volume had a significant effect on PDI (*p* < 0.05) in all formulations except F3 and F4. Interestingly, PDI in these two formulations was higher than 0.250, which already raised concerns regarding their stability. In order to evaluate the effect of beads volume after one hour of milling solely, one-way ANOVA was conducted and revealed significant influence in formulations F1, F6, and F8, but after performing post hoc tests, the significant difference was noticed only when comparing 20% and 60% bead volumes (F6 and F8) or 20% and 40% (F1). Indeed, when analyzing PDI values, the results were inconsistent, so no general conclusion can be drawn for all formulations. For example, in formulations F1, and F5, where the stabilizer ratio was 1:10, PDI was the highest when 20% beads volume was used. On the contrary, in formulations where the stabilizer ratio was 1:4, the highest PDI was obtained when 60% beads volume was used. It could be speculated that in this case, with more beads, more breakages occurred, which led to the formation of the population of smaller drug particles that boarded the particle size distribution. In formulations F3 and F4, with the PDI values 0.250 or higher, the best results were obtained when 40% beads volume was used.

Considering that the goal of this part of the research was to find the best conditions to obtain dispersions with small particle size and acceptable size distribution, it was concluded that the milling time should be one hour. When choosing the beads amount, because of generally subtle differences between formulations made when using 60% and 40% beads volume, 40% was chosen for further research, since in all formulations, suitable results of particle size and size distribution were derived when this condition was used. Additionally, the technical advantage of using a smaller amount of beads is the smaller loss of dispersion during the separation process.

#### 3.2.2. Formulation Parameters

For nanocrystal stabilization, surfactants, polymers, or their combinations can be used. The benefit of combining the two stabilization mechanisms to achieve a stable colloidal dispersion is explained through the reduction in the self-repulsion between the ionic surfactant molecules in the presence of steric stabilizers, leading to closer packing of the molecules [17]. SLS was used in concentration below the critical micelle concentration, thus not influencing DK-I-60-3 solubility, as recommended for successful stabilization [18]. Indeed, the solubility of DK-I-60-3 in medium containing SLS and PVP in concentrations as in F5, F6, and F8 was 5.52 ± 1.22, 3.47 ± 0.25, and 3.05 ± 0.82 µg/mL, respectively, i.e., similar to the solubility in water. As for the polymeric stabilizers, molecular weight is an important factor in overcoming the van der Waals forces, and on the other side preventing aggregation upon particle bridging. The general recommendation is to use polymeric chains from approximately 5000 to 25 000 g/mol [8].

When comparing the particle size and size distribution in formulations with different SLS concentrations but the same SLS: PVP ratio (Table 3), particle size was significantly smaller (*p* < 0.05, Student *t*-test) when 0.05% SLS was used, except in the case of 1:4 ratio, where the difference in particle size was only 10 nm. It is well known that with reducing particle size to the nanometer range, the surface-to-volume ratio increases dramatically, so obtained nanoparticles are prone to aggregation to hide exposed hydrophobic surfaces. For that reason, amphiphilic substances or polymers in proper concentrations are added to cover these regions and ensure the preservation of small particle size [19]. If the stabilizer concentration is insufficient, the drug particles cannot be effectively coated, while excessive stabilizers concentration could lead to particle aggregation and bridging due to the attachment of several drug particles to the same stabilizer molecule [18]. It was clear that stabilizers’ concentration in formulations F1–F4 was not enough to ensure particle stabilization, which resulted in higher particle size after preparation. When the stabilizers’ concentration was increased, in formulations F5–F8, particle size after preparation was below 200 nm, with a relatively narrow distribution (PDI < 0.250).

Zeta potential measurements provided insight into the nanocrystal particle coverage. In formulations F1–F4, with lower concentrations of the stabilizers, absolute zeta potential values were low regardless of the SLS:PVP ratio. The zeta potential of particles in formulation F5 was also around −20 mV; however, with the SLS:PVP ratio increase, the absolute zeta potential values increased as well (Table 3). The layer of adsorbed polymer molecules on the particle surface moves the shear plane to a longer distance, resulting in lower zeta potential [20]. When the polymer concentration is low, the particle surface is not densely covered by the polymer, and the anionic surfactant can more easily reach the surface of the nanocrystal. Consequently, the zeta potential increases with the increase in the SLS portion [8].

#### 3.2.3. Stability Study

Particle size and zeta potential of the formulations F5–F8 were checked after one and three months of storage at room temperature. Compared to initial values, particle size after one month of storage increased by around 10 nm, and then the values stayed unchanged for an additional two months (Table 4). The exception is formulation F7, where the change in particle size was more pronounced (around 40 nm), with a significant change in polydispersity index (F(2,6) = 8.951, *p* < 0.05, ANOVA). The stability study demonstrated the significance of PVP as a stabilizer, as well as the importance of balanced SLS and PVP concentration for particle size preservation. However, solidification of developed dispersions would be the next step to maintain stability. Besides for storage, this process would be important for dose-ranging studies, which are mandatory for new drug substances [2,9].

#### 3.2.4. Atomic Force Microscopy (AFM)

**Figure 3:**
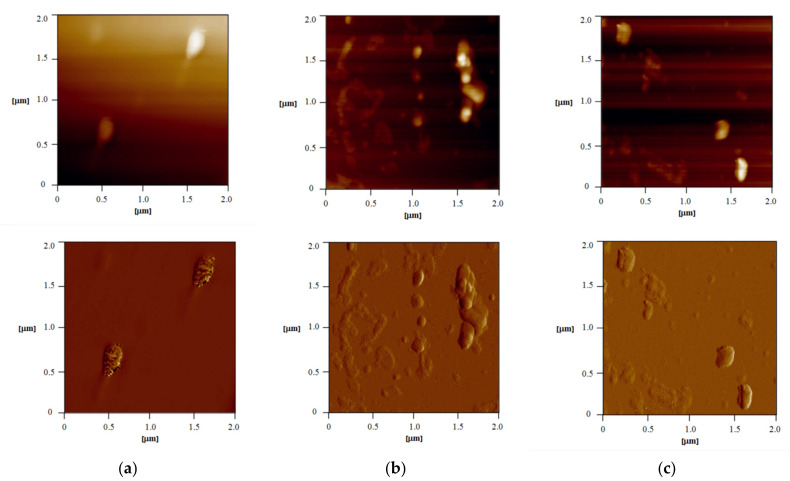
AFM images of samples F5 (**a**), F6 (**b**), and F8 (**c**): 2D topography of 2 × 2 μm area of the sample (up) and error signal of 2 × 2 μm area of the sample (down).

Although a very powerful method for particle size determination, dynamic light scattering (DLS) is limited to spherical objects, as it provides information about hydrodynamic radius [21]. Moreover, although it is suitable to detect major issues with sample integrity and stability because of the low resolution, it is not applicable as the only method for the determination of particle size and polydispersity of nanoparticles [22]. Therefore, for samples in which non-spherical particles are expected, visualization by some microscopic technique is significant for the right interpretation of the results. Atomic force microscopy is considered a valuable tool for nanocrystal characterization as it can provide information about the shape and structure of the particles in nano-range resolution [18]. AFM images of samples F5, F6, and F8 are presented in Figure 3. Nanocrystals were of uniform size and shape, and the size was in suitable correlation with the DLS results (Table 3). In the first sample, F5 (Figure 3a), particles were well dispersed and with dimensions of around 180 nm (width), 400 nm (length), and 40 nm (height). In sample F6 (Figure 3b), individual particles, as well as aggregates (probably formed during sample preparation), could be seen. Particles were around 130 nm in width. A similarity was noticed in F8 (Figure 3c).

#### 3.2.5. Differential Scanning Calorimetry (DSC) and X-ray Powder Diffraction Analysis (XRPD)

**Figure 4:**
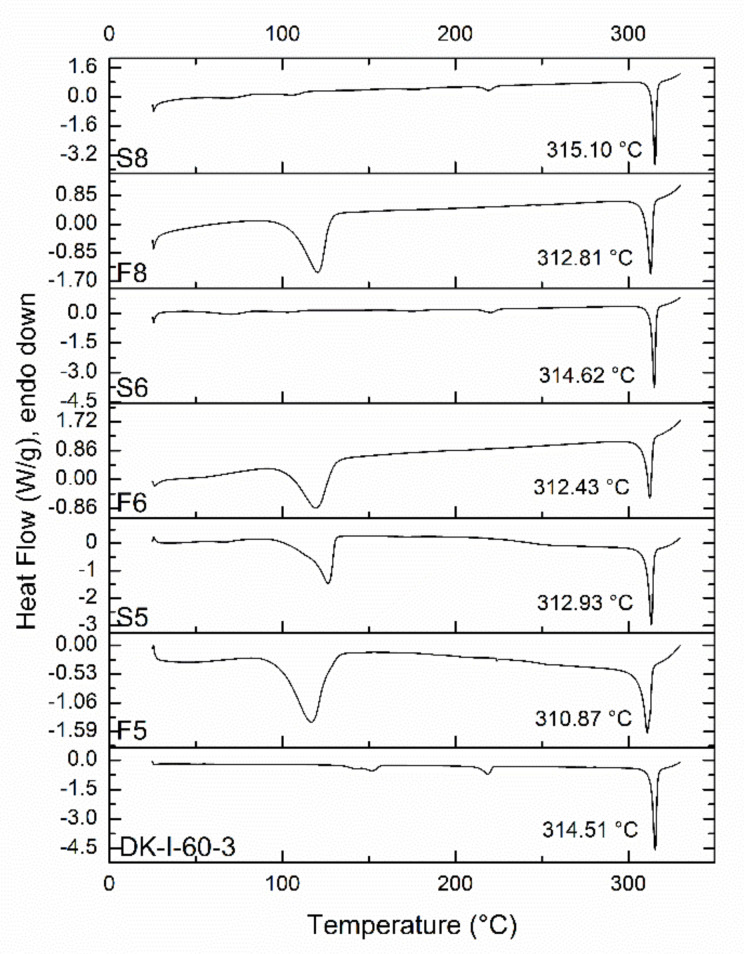
DSC thermograms of unprocessed DK-I-60-3, nanocrystal dispersions (F5, F6, and F8), and corresponding suspensions (S5, S6, and S8).

Results from DSC analysis are presented in Figure 4. Being a crystalline substance, DK-I-60-3 quickly melts upon heating, with a sharp peak visible on the DSC thermogram. The peak temperature in DK-I-60-3 was around 314 °C, and similar results were obtained in the case of physical mixtures S6 and S8. The melting peak in physical mixture S5 was 2 °C lower, probably due to interactions between DK-I-60-3 and excipients, as their concentration was the highest in this formulation. The melting peak in nanocrystalline formulations F5, F6, and F8 was around 2 °C degrees lower when compared to the corresponding physical mixtures, which can be explained by smaller particle sizes [23]. Melting point depression could also be connected to the formation of crystal lattice defects during wet ball milling, as it is a high-energy process [24]. The additional broad peak at 120 °C in thermograms of samples S5, F5, F6, and F8 could be associated with residual water and PVP.

**Figure 5:**
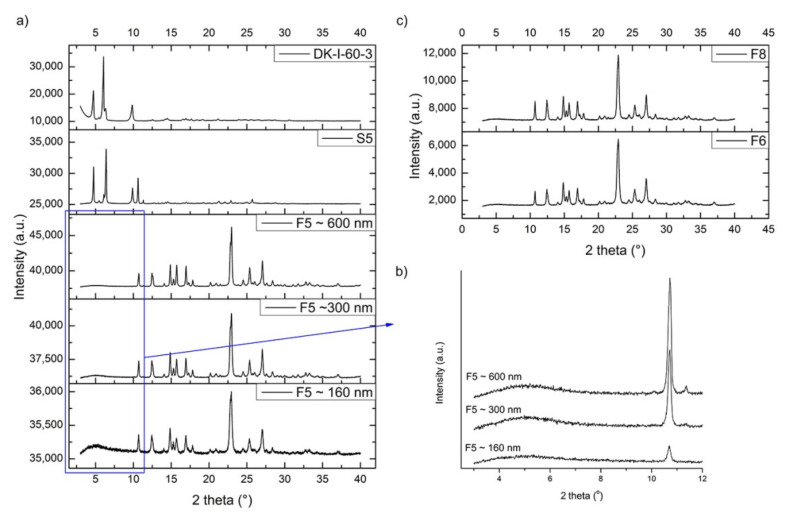
(**a**) XRPD patterns of unprocessed DK-I-60-3, suspension S5 and nanocrystal dispersion F5 with different particle size; (**b**) diffractograms of nanocrystal dispersion F5 with different particle 3–12° 2 theta; (**c**) XRPD patterns of nanocrystal dispersions F6 and F8.

To obtain a more detailed insight into the physical state of obtained nanocrystals, XRPD was conducted. Results showed that sample S5 (suspension that corresponds to formulation F5) predominantly contained crystalline DK-I-60-3 but not SLS, as can be seen from Figure 5a. This can be explained by SLS being in an amorphous state or lost during sample preparation due to incomplete attachment on the particle surface. Reflection intensity changes for DK-I-60-3 crystals in coarse powder and S5 could be ascribed to preferential orientation difference of DK-I-60-3 crystals (prismatic to needle-like, Figure 1a) in two samples. The milling process reduced average crystallite size and induced crystal defects concentration increase in F5, which is typical for nanocrystals [25]. Consequently, with prolonged milling and reduced particle size, the reflections on XRPD diagrams were broadened [26]. Intensive reflections below 10° 2 theta lost a great amount of intensity in nanocrystal samples. However, quite a broad reflection centered at about 5° 2 theta could be noticed in samples with different particle sizes. As it can be seen from Figure 5b, with the milling intensity increase, this broad reflection intensity decreased, indicating that nanonization was quite an intensive process. A similarity was noticed for F6 and F8, Figure 5c.

#### 3.2.6. Dissolution Study and Solubility of Nanocrystals

**Figure 6:**
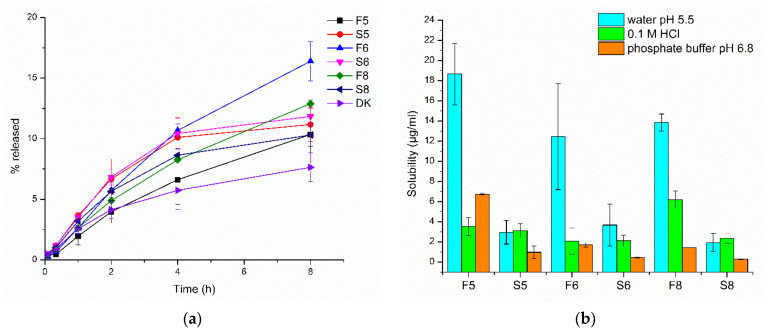
(**a**) Dissolution profiles of nanocrystal dispersions (F5, F6, and F8), corresponding suspensions (S5, S6, and S8) and coarse DK-I-60-3 (*p* < 0.05: F5 vs. S5 (20 min), F5 vs. S5 (60 min); F6 vs. S6 (20 min), F6 vs. S6 (60 min), F6 vs. S6 (8 h); F8 vs. S8 (60 min), F8 vs. S8 (2 h), F8 vs. S8 (8 h); F5 vs. F6 (4 h), F5 vs. F6 (8 h); F6 vs. F8 (4 h), F6 vs. F8 (8 h); F5 vs. DK (20 min); F6 vs. DK (20 min), F6 vs. DK (2 h), F6 vs. DK (4 h), F6 vs. DK (8 h); F8 vs. DK (8 h), Univariate ANOVA); (**b**) solubility of DK-I-60-3 in nanocrystal dispersions (F5, F6, and F8) and corresponding suspensions (S5, S6, and S8) in different media at 37 °C.

Dissolution profiles of nanocrystal dispersions (F5, F6, and F8), the corresponding suspensions (S5, S6, and S8), as well as coarse DK-I-60-3 are shown in Figure 6a. The DK-I-60-3 content in nanocrystalline formulations F5, F6, and F8 was 93.79 ± 2.86%, 94.89 ± 1.36%, and 92.32 ± 2.99%, respectively, relative to the theoretical concentration. It can be noted that the differences between the nanocrystals formulations and suspensions were not as pronounced as expected according to the Noyes–Whitney equation. The reason for the obtained results could be found in dialysis membrane acting as a permeation barrier or very low solubility in the dissolution medium (17.12 ± 0.07 µg/mL) [27]. The particle size of nanocrystals in the dialysis bag after the experiment did not change (*p* > 0.05, Student *t*-test, Table S2), confirming the strong influence of dialysis membrane on dissolution. Therefore, the dialysis membrane method most probably did not give a true release profile, which was previously warned by Moreno-Bautista and Tam [28] for colloid systems. However, it could be used for formulation comparisons. Although only the discrete differences could be observed between the dissolution profiles in Figure 6a, there was an obviously different trend in dissolution kinetics between nanocrystalline dispersions and physical mixtures, while in suspensions, the plateau was reached after 4 h, and the dissolution rate of nanocrystals remained unchanged.

Solubility of DK-I-60-3 in nanocrystal dispersions as well as in corresponding suspensions was determined in water, 0.1 M hydrochloride acid, and phosphate buffer (pH 6.8). In preformulation studies, it was noticed that the solubility of coarse DK-I-60-3 did not depend on pH value (Table 2), and this was similarly confirmed in these experiments in suspension samples. On the other hand, the solubility of nanocrystals changed in different media. In ultra-purified water, the solubility was three to nine folds higher (Figure 6b) in comparison to suspension with the same composition (*p* < 0.05, Student *t*-test). Similar results were published for nanosuspensions of cefdinir [29]. An increase in saturation solubility is expected according to the Ostwald–Freundlich equation [30], due to reduced particle size but also as a consequence of changes in crystal lattice energy [29].

In the other two media, however, the solubility decreased significantly, which indicated stability impairment due to low pH value and/or high ionic strength (Figure 6b). Electrostatic repulsion of nanocrystals can be inhibited by high acid concentrations, leading to particle agglomeration [18]. It was interesting that the solubility in 0.1 M HCl in nanosuspensions decreased to the values similar to the ones in the corresponding suspensions (*p* = 0.563 and 0.965 for F5 vs. S5, and F6 vs. S6, respectively, Student *t*-test) except in the case of F9 (*p* < 0.01, Student *t*-test). In this formulation, the solubility of DK-I-60-3 in 0.1 M HCl was surprisingly high. The highest solubility in phosphate buffer was achieved in the F5 formulation with the highest PVP concentration. Polymeric stabilizers are considered advantageous to decrease the influence of ions on nanocrystals’ stability [31].

#### 3.2.7. In Vivo Pharmacokinetic and Biodistribution Studies

**Figure 7:**
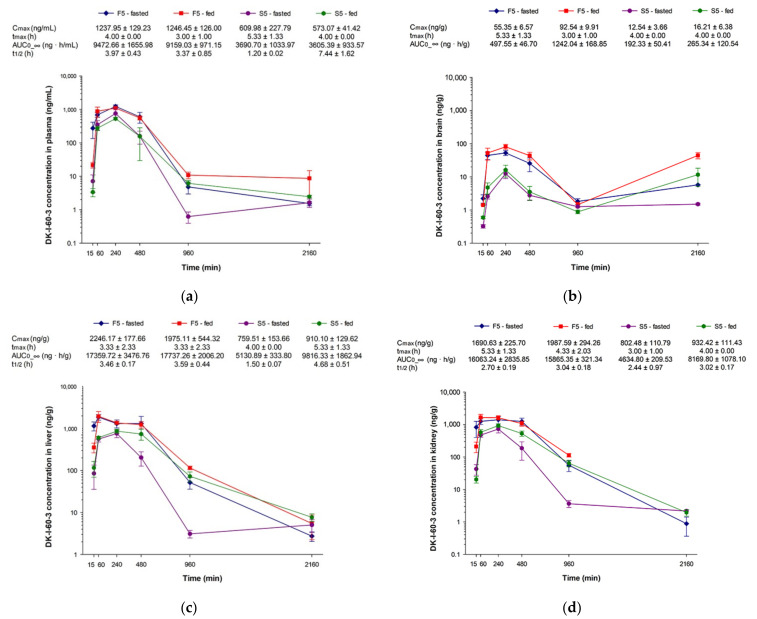
Concentration versus time curves and calculated pharmacokinetic parameters of nanocrystal dispersion (F5) and suspension (S5) in (**a**) plasma, (**b**) brain, (**c**) liver, and (**d**) kidney after oral administration in rats (n = 3 per time point) at a dose of 10 mg/kg, in fasted and fed state (C_max_—maximum concentration, t_max_—time of maximum concentration, t_1/2_—terminal elimination half-life, AUC_0-__∞_—area under the concentration versus time curve). Terminal elimination half-life in the brain was not calculated due to erratic concentration-time profiles.

Concentration-time curves for plasma, brain, liver, and kidney after administration of nanocrystal dispersion (F5) or corresponding suspension (S5) in fasted or fed rats are presented in Figure 7. As it can be noticed, after suspension administration, maximum concentration (C_max_) and area under the curve (AUC) in plasma were similar in the fasted and fed state (*p* = 0.998 and 0.100 for C_max_ and AUC, respectively, Student *t*-test). In investigated solid tissues, although average values of AUC seem to be different, even two times higher in the group of fed animals, statistically the differences were not significant (*p* = 0.933, 0.484, and 0.719 for brain, liver, and kidney, respectively, Student *t*-test), due to interindividual differences. Calculated total AUC in the groups of animals in the fed state was 1.5 times higher compared to total AUC in fasted state animals, probably because of the smaller elimination rate. On the other hand, in both groups of animals that received nanocrystal dispersion, all calculated pharmacokinetic parameters were similar with smaller standard deviations, indicating reduced variations in DK-I-60-3 absorption. Similar observations of the role of nanocrystals in the mitigation of interindividual differences were confirmed in the case of itraconasole nanosuspensions [32]. More balanced absorption after nanocrystals could be a consequence of small and uniformed particle size, causing more or less the same biodistribution afterward in both fasted and fed groups.

When comparing different treatments, nanocrystal and coarse dispersion, C_max_ and AUC in all investigated matrices were two to three times higher after nanocrystals regardless of food intake (Figure 7). Significant differences in plasma AUC following different treatments (*p* < 0.05, ANOVA) proved the increased extent of absorption following nanocrystal administration. Although results of the nanocrystals’ solubility (Figure 6b) did not promise improved pharmacokinetics, other factors such as permeability enhancement could give rise to better absorption [33]. Moreover, the pH value of the empty and fed stomach in rats is 4–5 [34], so the solubility decrease would not be as prominent as first expected. It is evident that, despite numerous in vivo studies, the way by which nanocrystals improve oral bioavailability remains unclear, and probably dissolution and endocytosis are acting simultaneously [35]. Additionally, the possible solubilization of DK-I-60-3 by the bile salts and the interaction between the bile salts and SLS could not be neglected [34]. The bioavailability increase in solid tissues was particularly important for brain delivery as a target organ. Although brain intake was in a small scope, the delivery was relatively fast, as the maximum concentrations were achieved shortly after in plasma. Relatively elevated concentrations in the brain after 36 h could indicate possible depot formations of DK-I-60-3 in the body. This caused the higher AUC in the brain following the F5 formulation in the fed state. The exact reason for the obtained results is still unknown but will be investigated in future research.

**Figure 8:**
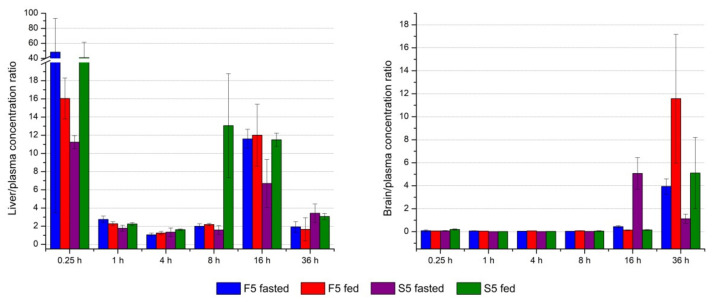
Ratio between DK-I-60-3 concentration in liver (**left**) and brain (**right**) in each time point.

The distribution of the drug after administration is mainly dependent on uptake competitions between blood and tissues, as well as competitions among individual tissues [36]. After calculating the ratio between concentrations in liver and plasma in each time point (Figure 8, left), it can be noticed that DK-I-60-3 has an affinity to retain in this organ (calculated ratio > 1). As expected, 15 min after administration, the highest concentrations were observed in the liver. The elimination from the liver was slower than from the kidney, for example, according to elimination half-time (Figure 7c,d), which could indicate retention of the substance in the liver to some extent. Forming depots in the liver could also be speculated from the expressively risen liver to plasma ratio 16 h after treatment. On the other side, due to the blood-brain barrier, concentrations in the brain were smaller than the ones in plasma in the first five time points (Figure 8, right). Yet, in the last time point, 36 h after administration, DK-I-60-3 concentration was two to three times higher, indicating slower elimination from this organ. The exception is the case of the suspension administered in the fasted state, where higher concentrations were observed after 16 h, after which they began to decrease. Therefore, at this point, we could not know for sure what caused the concentration to rise at the last time point in the brain. This could be the consequence of erratic or slower distribution, depot effect in the body, potential interaction with the brain lipids, or the combination of several factors. The structure of the blood-brain barrier is another piece of the complex puzzle when a drug is intended for brain delivery.

It was interesting that similar concentration-time profiles in the brain were noticed for both formulations (nanocrystal dispersions and suspensions), which was not the case in our previous research with the monodeuterated analog DK-I-56-1 [13]. In the mentioned research, after intraperitoneal administration of suspension, the biphasic concentration-time profile was obtained. In the brain after nanosuspension and solution, the concentrations 36 h after administration were a little elevated as well, compared to the concentrations in the 16 h time point. On the other hand, in Knutson et al. [12], the concentrations in the brain at the 24 h time point after intravenous and oral administration of DK-I-56-1 nanoemulsion were higher than the ones in plasma. It seems that the observed higher levels in the brain in the time points later than 16 h after administration are the consequence of the properties of the substance, rather than formulations used.

## 4. Conclusions

Being a brick dust substance prone to crystalize after melting, DK-I-60-3 was characterized as a suitable candidate for nanonization. Nanocrystal dispersions of DK-I-60-3 with small particle size and three months’ stability were prepared by the modified wet media milling technique. Content of DK-I-60-3 in developed dispersions close to theoretical values indicated no significant adsorption of DK-I-60-3 to milling beads, while XRPD proved no impurities from the material of the used beads in dispersions. Process and formulation parameters had a significant influence on physicochemical characteristics of developed dispersions. It was proven that balanced concentrations of SLS and PVP are necessary for nanocrystal stabilization. Besides solubility enhancement, particle size reduction induced a crystallite size decrease and the formation of crystal defects.

The pharmacokinetic study provided an interesting insight into the DK-I-60-3 fate after oral administration. The prandial state did not have a major influence on absorption, although variations were more prominent after suspension administration. Overall bioavailability was increased after nanocrystal dispersion; however, a smaller portion of DK-I-60-3 has reached the brain. Because of very low stabilizer concentration, which did not change solubility nor other physical properties of DK-I-60-3, it is possible that the results from this study reflected inherent pharmacokinetic properties of DK-I-60-3. Therefore, the results from the performed research demonstrated the improvement of challenging physical characteristics of the drug substance by nanocrystal technology, as well as enhancement of bioavailability after oral administration of developed nanocrystals.

## Figures and Tables

**Table 1:** Composition of developed nanocrystal dispersions.

Formulation	DK-I-60-3 (% *w*/*w*)	SLS (% *w*/*w*)	PVP (% *w*/*w*)	Water (% *w*/*w*)	SLS:PVP Ratio
F1	0.20	0.02	0.20	to 100	1:10
F2	0.20	0.02	0.08	to 100	1:4
F3	0.20	0.02	0.04	to 100	1:2
F4	0.20	0.02	0.02	to 100	1:1
F5	0.20	0.05	0.50	to 100	1:10
F6	0.20	0.05	0.20	to 100	1:4
F7	0.20	0.05	0.10	to 100	1:2
F8	0.20	0.05	0.05	to 100	1:1

**Table 2:** Solubility of DK-I-60-3 in investigated solvents (mean ± SD, n = 3).

Solvent	Solubility (µg/mL)
Water (pH 5.23)	6.34 ± 1.14
0.1 M HCl (pH 1.2)	7.21 ± 0.40
Phosphate buffer (pH 6.8)	7.75 ± 1.09
Medium-chain tryglycerides	50.33 ± 7.19
Soybean oil	13.81 ± 0.69
Castor oil	57.96 ± 1.79
Benzyl alcohol	10,373.53 ± 371.41
Polyethylene glycol 400	8224.72 ± 103.20
Isopropanol	1009.41 ± 19.50
Methanol	802.86 ± 103.43
Ethanol, 96%, *v*/*v*	1807.70 ± 21.67
Dimethyl sulfoxide	166,495.77 ± 4075.05

**Table 3:** Particle size (z-ave), polydispersity index (PDI), and zeta potential (ZP) of nanocrystal dispersions after one hour of milling (mean ± SD, *n* = 3).

Formulation	Milling Media Volume (%, *v*/*v*)	z-Ave (nm)	PDI	ZP (mV)
	60	195.8 ± 1.7	0.238 ± 0.029	
F1	40	236.7 ± 1.9	0.208 ± 0.002	−17.5 ± 1.6
	20	233.6 ± 2.7	0.260 ± 0.007	
F2	60	137.7 ± 2.2	0.226 ± 0.015	
	40	165.5 ± 4.3	0.226 ± 0.011	−16.8 ± 0.5
	20	229.9 ± 4.9	0.205 ± 0.012	
F3	60	235.1 ± 4.2	0.273 ± 0.044	
	40	239.3 ± 3.3	0.251 ± 0.017	−28.0 ± 1.2
	20	298.6 ± 9.1	0.301 ± 0.019	
F4	60	155.6 ± 2.3	0.258 ± 0.040	
	40	176.0 ± 3.1	0.250 ± 0.040	−19.2 ± 0.7
	20	233.1 ± 6.2	0.277 ± 0.016	
F5	60	148.7 ± 2.5	0.231 ± 0.010	
	40	174.1 ± 0.6	0.231 ± 0.015	−21.8 ± 5.6
	20	222.8 ± 3.3	0.253 ± 0.021	
F6	60	138.7 ± 2.3	0.225 ± 0.011	
	40	153.1 ± 1.8	0.197 ± 0.021	−31.9 ± 1.8
	20	185.3 ± 1.5	0.179 ± 0.012	
F7	60	151.6 ± 4.3	0.206 ± 0.012	
	40	170.2 ± 2.4	0.220 ± 0.008	−36.8 ± 1.1
	20	214.2 ± 5.5	0.205 ± 0.022	
F8	60	143.6 ± 1.4	0.231 ± 0.006	
	40	160.4 ± 3.1	0.208 ± 0.014	−38.1 ± 1.9
	20	182.7 ± 1.2	0.202 ± 0.011	

**Table 4:** Particle size (z-ave), polydispersity index (PDI), and zeta potential (ZP) of nanocrystal dispersions after one and three months of storage (mean ± SD, n = 3).

Formulation	z-Ave (nm)		PDI		ZP (mV)	
	1 Month	3 Months	1 Month	3 Months	1 Month	3 Months
F5	183.5 ± 4.2	181.2 ± 3.3	0.227 ± 0.024	0.212 ± 0.008	−18.0 ± 0.3	−20.7 ± 1.5
F6	162.2 ± 4.2	161.8 ± 5.1	0.204 ± 0.007	0.212 ± 0.010	−35.3 ± 1.2	−30.4 ± 0.8
F7	212.0 ± 5.3	214.2 ± 5.9	0.238 ± 0.004	0.220 ± 0.005	−36.7 ± 1.1	−40.7 ± 1.1
F8	175.4 ± 4.1	175.8 ± 5.2	0.208 ± 0.021	0.257 ± 0.081	−41.8 ± 2.6	−38.3 ± 1.0

## Supplementary Materials

The following are available online at https://www.mdpi.com/article/10.3390/pharmaceutics13081188/s1, Table S1: Particle size (z-ave) and polydispersity index (PDI) of nanocrystal dispersions after 30 min of milling (mean ± SD, n = 3), Table S2: Particle size (z-ave) and polydispersity index (PDI) before and after dissolution study (mean ± SD, n = 3).
