# Supplementary material for: Overcoming the Low Oral Bioavailability of Deuterated Pyrazoloquinolinone Ligand DK-I-60-3 by Nanonization: A Knowledge-Based Approach
Source: Pharmaceutics. 2021 Jul 31;13(8):1188. doi: 10.3390/pharmaceutics13081188 (PMC8400889; doi:10.3390/pharmaceutics13081188)
Supplement: Supplementary file 1 [file pharmaceutics-13-01188-s001.zip › pharmaceutics-1285136-supplementary.pdf]

pharmaceutics

MDPI

Supplementary Materials: Overcoming the Low Oral Bioavailability of Deuterated Pyrazoloquinolinone Ligand DK-I-60-3 by Nanonization: A Knowledge-Based Approach

Jelena R. Mitrović, Branka Divović-Matović, Daniel E. Knutson, Jelena B. Đoković, Aleksandar Kremenović, Vladimir D. Dobričić, Danijela V. Randjelović, Ivana Pantelić, James M. Cook, Miroslav M. Savić and Snežana D. Savić

Table S1. Particle size (z-ave) and polydispersity index (PDI) of nanocrystal dispersions after 30 min of milling (mean ± SD, n = 3).

Formulation	Milling media volume (%, *v/v*)	z-ave (nm)	PDI
F1	60	239.5 ± 4.0	0.253 ± 0.018
	40	282.5 ± 5.9	0.287 ± 0.027
	20	308.9 ± 5.9	0.408 ± 0.052
F2	60	186.9 ± 3.6	0.224 ± 0.033
	40	232.8 ± 4.2	0.229 ± 0.020
	20	309.6 ± 12.1	0.299 ± 0.032
F3	60	348.0 ± 7.5	0.341 ± 0.009
	40	382.2 ± 2.8	0.374 ± 0.032
	20	425.1 ± 11.7	0.441 ± 0.016
F4	60	197.1 ± 4.1	0.274 ± 0.007
	40	231.5 ± 4.5	0.287 ± 0.057
	20	322.6 ± 7.9	0.348 ± 0.032
F5	60	190.4 ± 1.3	0.230 ± 0.022
	40	218.4 ± 0.7	0.271 ± 0.017
	20	284.7 ± 1.6	0.318 ± 0.028
F6	60	182.8 ± 2.8	0.181 ± 0.017
	40	196.4 ± 3.5	0.186 ± 0.017
	20	237.3 ± 4.6	0.194 ± 0.003
F7	60	198.0 ± 5.1	0.211 ± 0.007
	40	227.2 ± 3.5	0.200 ± 0.016
	20	278.3 ± 6.2	0.274 ± 0.009
F8	60	188.7 ± 3.8	0.210 ± 0.015
	40	211.1 ± 2.5	0.224 ± 0.012
	20	242.9 ± 1.8	0.217 ± 0.014

Table S2. Particle size (z-ave) and polydispersity index (PDI) before and after dissolution study (mean ± SD, n = 3).

Formulation	z-ave (nm)		PDI	
	Before dissolution	After dissolution	Before dissolution	After dissolution
F5	164.1 ± 5.2	166.7 ± 2.8	0.208 ± 0.013	0.195 ± 0.014
F6	150.5 ± 3.1	147.9 ± 0.4	0.196 ± 0.007	0.236 ± 0.009
F8	176.4 ± 6.1	186.3 ± 1.6	0.186 ± 0.012	0.185 ± 0.015
